# Supplementary material for: The target of the DEAH-box NTP triphosphatase Prp43 in Saccharomyces cerevisiae spliceosomes is the U2 snRNP-intron interaction
Source: eLife. 2016 Apr 26;5:e15564. doi: 10.7554/eLife.15564 (PMC4866824; doi:10.7554/eLife.15564)
Supplement: Figure 2—source data 1. — Every two fractions from the glycerol gradient shown in Figure 2C were pooled, and proteins were recovered and separated by PAGE. Proteins were cut out of the gel, digested in-gel with trypsin and extracted. The extracted peptides were analyzed in a liquid-chromatography-coupled electrospray ionization orbitrap mass spectrometer (LTQ Orbitrap XL) under standard conditions. Proteins were identified by searching fragment spectra against the yeast S. cerevisiae Genomic Database (SGD), using Mascot as a search engine. Numbers represent the absolute number of peptides sequenced for a protein found in a particular preparation. The table contains information about the S. cerevisiae protein and the calculated molecular weight in kilodaltons. Proteins are grouped in organizational and/or functional subgroups. The peaks in the gradient of the U2 and 35S U5 snRNPs and their associated proteins are marked in green and orange, respectively. DOI: http://dx.doi.org/10.7554/eLife.15564.005 [file elife-15564-fig2-data1.docx]

| **Yeast Protein name** | **MW [kDa]** | **Number of peptides sequenced** | | | | | | | | |
| --- | --- | --- | --- | --- | --- | --- | --- | --- | --- | --- |
| **Column** | | **1** | **2** | **3** | **4** | **5** | **6** | **7** | **8** | **9** |
| **BactΔPrp2 spliceosomes supplemented with** | | **Prp43_Ntr1GP + ATP** | | | | | | | | |
| **Analysis with Orbitrap device** | | **same volume** | | | | | | | | |
| **Gradient fractions analyzed** | | **1+2** | **3+4** | **5+6** | **7+8** | **9+10** | **11+12** | **13+14** | **15+16** | **17+18** |
| **Sm proteins** | | | | | | | | | | |
| B | 22.4 |  | 8 | 1 |  | 3 | 7 | 6 | 6 | 8 |
| D1 | 16.3 |  | 6 | 3 | 3 | 6 | 9 | 10 | 8 | 11 |
| D2 | 12.8 | 3 | 7 | 8 | 3 | 7 | 9 | 10 | 9 | 11 |
| D3 | 11.2 | 1 | 6 | 6 | 4 | 7 | 13 | 9 | 8 | 16 |
| E | 10.4 |  |  | 1 |  | 1 | 4 | 3 | 3 | 4 |
| F | 9.6 |  | 2 | 2 | 1 | 2 | 3 | 3 | 2 | 3 |
| G | 8.5 | 1 | 2 | 3 | 2 | 5 | 7 | 5 | 5 | 7 |
| **U2 snRNP proteins** | | | | | | | | | | |
| Rse1 | 153.8 |  |  | 1 | 34 | 86 | 116 | 93 | 54 | 79 |
| Hsh155 | 110 |  |  |  | 38 | 72 | 86 | 63 | 49 | 67 |
| Prp9 | 63 |  | 2 | 12 | 39 | 50 | 57 | 51 | 32 | 37 |
| Cus1 | 50.2 |  |  |  | 8 | 24 | 52 | 31 | 19 | 24 |
| Prp21 | 33 |  | 2 | 8 | 16 | 20 | 36 | 24 | 15 | 27 |
| Prp11 | 29.9 | 1 | 2 | 2 | 7 | 12 | 18 | 12 | 6 | 17 |
| Lea1 | 27.2 | 16 | 25 | 17 | 18 | 25 | 31 | 17 | 6 | 21 |
| Hsh49 | 24.5 |  |  |  | 3 | 7 | 8 | 6 | 4 | 7 |
| Msl1 | 12.8 | 5 | 8 | 4 | 2 | 5 | 11 | 7 | 3 | 7 |
| Rds3 | 12.3 |  |  |  |  | 5 | 7 | 4 | 2 | 5 |
| Ysf3 | 10 |  |  |  |  | 2 | 3 | 3 | 2 | 2 |
| **U5 snRNP proteins** | | | | | | | | | | |
| Prp8 | 279.5 |  |  | 1 | 40 | 80 | 140 | 165 | 145 | 178 |
| Brr2 | 246.2 |  | 75 | 100 | 100 | 102 | 139 | 139 | 116 | 141 |
| Snu114 | 114 |  |  | 2 | 32 | 59 | 75 | 98 | 96 | 104 |
| **NTC/Prp19 complex proteins** | | | | | | | | | | |
| Syf1/ Ntc90 | 100 | 5 | 14 | 9 | 2 |  | 22 | 50 | 58 | 62 |
| Clf1/ Ntc77 | 82.4 |  | 6 | 2 | 1 |  | 18 | 35 | 41 | 54 |
| Cef1/ Ntc85 | 68 |  |  |  |  |  | 17 | 34 | 31 | 44 |
| Prp19 | 56.6 |  | 6 | 15 | 10 | 6 | 28 | 44 | 40 | 43 |
| Isy1/ Ntc30 | 28 |  | 2 | 1 |  |  | 5 | 9 | 7 | 13 |
| Syf2/ Ntc31 | 25 | 4 | 4 | 4 | 4 | 4 | 6 | 11 | 10 | 14 |
| Snt309/ Ntc25 | 21 |  |  |  |  |  | 1 | 10 | 6 | 11 |
| Ntc20 | 16 | 1 |  |  |  |  | 4 | 7 | 5 | 7 |
| **NTC related proteins** | | | | | | | | | | |
| Prp46 | 51 | 8 | 14 | 11 | 5 | 1 | 14 | 27 | 30 | 38 |
| Prp45 | 42.5 | 22 | 22 | 18 | 11 | 3 | 23 | 39 | 29 | 37 |
| Ecm2 | 41 | 14 | 6 | 1 |  |  |  | 2 | 1 | 1 |
| Cwc2 | 38.4 | 23 | 22 | 12 | 6 | 1 | 10 | 22 | 19 | 25 |
| Bud31/ Cwc14 | 18.4 | 4 | 5 | 2 | 1 | 1 | 5 | 6 | 4 | 5 |
| Cwc15 | 20 | 19 | 19 | 17 | 17 | 13 | 17 | 13 | 4 | 10 |
| Prp17/ Cdc40 | 52 | 14 | 19 | 11 | 6 | 3 | 17 | 22 | 14 | 23 |
| Yju2/ Cwc16 | 32 | 1 |  |  |  |  | 1 | 12 | 12 | 21 |
| Cwc21 | 15.7 | 9 | 7 | 5 | 4 | 1 | 4 | 2 | 1 | 4 |
| Cwc22 | 67.3 | 28 | 21 | 12 | 1 | 1 | 8 | 5 | 5 | 5 |
| Cwc27 | 35 | 30 | 28 | 22 | 9 | 1 | 4 | 1 |  |  |
| **RES complex proteins** | | | | | | | | | | |
| Bud13 | 30.5 | 27 | 30 | 24 | 21 | 15 | 20 | 13 | 8 | 12 |
| Pml1 | 12.6 | 15 | 19 | 17 | 17 | 11 | 11 | 4 |  | 6 |
| Ist3/Snu17 | 17 | 9 | 10 | 8 | 8 | 7 | 9 | 8 | 1 | 3 |
