# Supplementary material for: The target of the DEAH-box NTP triphosphatase Prp43 in Saccharomyces cerevisiae spliceosomes is the U2 snRNP-intron interaction
Source: eLife. 2016 Apr 26;5:e15564. doi: 10.7554/eLife.15564 (PMC4866824; doi:10.7554/eLife.15564)
Supplement: Figure 3—source data 1. — Every three fractions from the glycerol gradient shown in Figure 3B were pooled, and proteins were recovered and separated by PAGE. Proteins were identified by mass spectrometry as above. Numbers represent the absolute number of peptides sequenced for a protein found in a particular preparation. The table contains information about the Saccharomyces cerevisiae protein and the calculated molecular weight in kilodaltons. Proteins are grouped in organizational and/or functional subgroups. The peaks in the gradient of the U2, U1 and tetra snRNPs and their associated proteins are marked in green, blue and yellow, respectively. DOI: http://dx.doi.org/10.7554/eLife.15564.007 [file elife-15564-fig3-data1.docx]

| **Yeast Protein name** | **MW [kDa]** | **Number of peptides sequenced** | | | | | |
| --- | --- | --- | --- | --- | --- | --- | --- |
| **Column** | | **1** | **2** | **3** | **4** | **5** | **6** |
| **B spliceosomes supplemented with** | | **Prp43_Ntr1GP + UTP** | | | | | |
| **Analysis with Orbitrap Device** | | **same volume** | | | | | |
| **Fractions** | | **1-3** | **4-6** | **7-9** | **10-12** | **13-15** | **16-18** |
| **Sm proteins** | | | | | | | |
| B | 22.4 | 10 | 19 | 20 | 28 | 39 | 10 |
| D1 | 16.3 | 1 | 25 | 31 | 41 | 42 | 22 |
| D2 | 12.8 | 5 | 22 | 36 | 31 | 51 | 20 |
| D3 | 11.2 | 8 | 15 | 26 | 43 | 52 | 34 |
| E | 10.4 |  | 1 | 5 | 10 | 7 | 2 |
| F | 9.6 |  | 5 | 5 | 7 | 10 | 4 |
| G | 8.5 | 2 | 6 | 15 | 21 | 17 | 12 |
| **U1 snRNP proteins** | | | | | | | |
| Prp39 | 74.7 |  |  | 26 | 60 | 38 | 24 |
| [Snu71](system:\Experiments%2006-07-08\Experiments%202006\splicing_database_MAC_MAY20\gene_names.xls#A688:O688) | 71.4 | 6 | 11 | 29 | 53 | 46 | 29 |
| Prp40 | 69.0 | 6 | 10 | 22 | 39 | 30 | 36 |
| Prp42 | 65.0 |  |  | 22 | 34 | 41 | 18 |
| Nam8 | 56.9 |  |  | 9 | 21 | 22 | 13 |
| Snu56 | 56.5 |  |  | 5 | 18 | 18 | 6 |
| Snp1 | 34.4 |  |  | 10 | 31 | 19 | 11 |
| Mud1 | 34.4 |  |  | 7 | 19 | 17 | 7 |
| Luc7 | 30.0 | 3 | 9 | 19 | 19 | 20 | 11 |
| Yhc1 | 27.0 |  |  | 6 | 11 | 15 | 5 |
| **U2 snRNP proteins** | | | | | | | |
| Rse1 | 153.8 |  | 42 | 208 | 192 | 251 | 126 |
| Hsh155 | 110.0 |  | 24 | 187 | 121 | 183 | 100 |
| Prp9 | 63.0 |  | 34 | 96 | 89 | 109 | 73 |
| Cus1 | 50.2 |  | 17 | 118 | 76 | 117 | 49 |
| Prp21 | 33.0 |  | 16 | 67 | 60 | 90 | 38 |
| Prp11 | 29.9 | 2 | 13 | 39 | 32 | 40 | 22 |
| Lea1 | 27.2 | 19 | 44 | 67 | 56 | 72 | 37 |
| Hsh49 | 24.5 | 1 | 7 | 25 | 19 | 22 | 12 |
| Msl1 | 12.8 | 4 | 18 | 26 | 21 | 29 | 10 |
| Rds3 | 12.3 |  | 4 | 14 | 12 | 18 | 8 |
| Ysf3 | 10.0 |  | 1 | 4 | 8 | 10 | 5 |
| **U5 snRNP proteins** | | | | | | | |
| Prp8 | 279.5 |  | 24 | 224 | 450 | 521 | 282 |
| Brr2 | 246.2 |  | 59 | 207 | 357 | 506 | 257 |
| Snu114 | 114.0 |  | 22 | 108 | 198 | 229 | 102 |
| Prp6 | 104.2 | 43 | 39 | 58 | 108 | 169 | 87 |
| **U4/U6 snRNP proteins** | | | | | | | |
| Prp31 | 56.3 | 16 | 19 | 36 | 48 | 84 | 26 |
| Prp3 | 56.0 | 4 | 18 | 34 | 54 | 79 | 36 |
| Prp4 | 52.4 | 5 | 16 | 30 | 71 | 74 | 59 |
| Snu13 | 13.6 | 1 | 6 | 4 | 7 | 14 | 5 |
| **U4/U6.U5 snRNP proteins** | | | | | | | |
| Snu66 | 66.4 | 3 | 13 | 74 | 132 | 160 | 84 |
| Spp381 | 34.0 | 5 | 2 | 22 | 45 | 57 | 19 |
| Prp38 | 28.0 | 6 | 4 | 12 | 30 | 29 | 17 |
| Snu23 | 23.0 | 5 | 14 | 24 | 51 | 64 | 35 |
| **LSm proteins** | | | | | | | |
| LSm2 | 11.2 | 3 | 7 | 10 | 25 | 33 | 15 |
| LSm3 | 10.0 | 2 | 2 | 1 | 7 | 8 | 5 |
| LSm4 | 21.3 | 4 | 15 | 10 | 20 | 33 | 20 |
| LSm5 | 10.4 | 2 | 4 | 5 | 9 | 9 | 6 |
| LSm6 | 9.4 | 2 | 5 | 6 | 11 | 11 | 16 |
| LSm7 | 13.0 | 2 | 8 | 8 | 11 | 17 | 11 |
| LSm8 | 12.4 |  | 7 | 5 | 12 | 15 | 6 |

Figure 3-source data 1a

| **Yeast Protein name** | **MW [kDa]** | **Number of peptides sequenced** | | | | | |
| --- | --- | --- | --- | --- | --- | --- | --- |
| **Column** | | **1** | **2** | **3** | **4** | **5** | **6** |
| **B spliceosomes supplemented with** | | **Prp43_Ntr1GP + UTP** | | | | | |
| **Analysis with Orbitrap Device** | | **same volume** | | | | | |
| **Fractions** | | **1-3** | **4-6** | **7-9** | **10-12** | **13-15** | **16-18** |
| **RES complex proteins** | | | | | | | |
| Bud13 | 30.5 | 27 | 52 | 74 | 50 | 46 | 14 |
| Pml1 | 23.6 | 19 | 23 | 19 | 23 | 16 | 5 |
| Ist3/Snu17 | 17.0 | 7 | 15 | 14 | 16 | 9 | 4 |
| **NTC/Prp19 complex proteins** | | | | | | | |
| Syf1/ Ntc90 | 100.0 | 20 | 40 | 44 | 49 | 44 | 41 |
| Clf1/ Ntc77 | 82.4 | 15 | 21 | 34 | 39 | 37 | 30 |
| Cef1/ Ntc85 | 68.0 |  | 19 | 30 | 34 | 40 | 22 |
| Prp19 | 56.6 | 7 | 49 | 74 | 58 | 90 | 70 |
| Isy1/ Ntc30 | 28.0 | 5 | 12 | 9 | 9 | 12 | 10 |
| Syf2/ Ntc31 | 25.0 |  | 3 | 5 | 9 | 11 | 9 |
| Snt309/ Ntc25 | 21.0 |  | 7 | 8 | 8 | 10 | 10 |
| Ntc20 | 16.0 | 3 | 5 | 5 | 6 | 7 | 6 |
| **NTC related proteins** | | | | | | | |
| Prp46 | 51.0 |  | 22 | 43 | 29 | 25 | 19 |
| Prp45 | 42.5 |  | 21 | 38 | 28 | 34 | 26 |
| Ecm2 | 41.0 |  |  |  |  |  |  |
| Cwc2 | 38.4 |  |  |  |  |  |  |
| Bud31/ Cwc14 | 18.4 | 2 | 2 | 9 | 9 | 9 | 4 |
| Cwc15 | 20.0 |  |  |  |  |  |  |

Figure 3-source data 1b
