# Supplementary material for: The target of the DEAH-box NTP triphosphatase Prp43 in Saccharomyces cerevisiae spliceosomes is the U2 snRNP-intron interaction
Source: eLife. 2016 Apr 26;5:e15564. doi: 10.7554/eLife.15564 (PMC4866824; doi:10.7554/eLife.15564)
Supplement: Figure 6—source data 1. — We used BS3, a chemical crosslinker with a spacer length of 11 Å, which is specific for lysines and N-termini. Data were analyzed by mass spectrometry and the spectra of Prp43_Ntr1GP interactions were selected. Spectra count indicates the number of times that one particular interaction was seen. The score indicates the probability of interaction between two proteins. High scores (i.e. ≥2) signify a higher confidence of crosslink identification. In the case of multiple spectra, only the highest score is reported. Three separate experiments were performed and are shown in Figure 6—source data 1a and b. (a) Proteins crosslinked to Prp43_Ntr1GP in affinity-purified Bact ΔPrp2 spliceosomes. Inter-molecular interactions between residues of Prp43_Ntr1GP and residues of the spliceosomal proteins indicated (i.e. Protein 2). (b) Intra-molecular interactions between residues (i.e. residue 1 and 2) of Prp43_Ntr1GP. DOI: http://dx.doi.org/10.7554/eLife.15564.012 [file elife-15564-fig6-data1.docx]

| **Prp43_**  **Ntr1GP**  **Residues** | **Protein 2**  **Residues** | **Experiment 1** | | **Experiment 2** | | **Experiment 3** | |
| --- | --- | --- | --- | --- | --- | --- | --- |
|  |  | **Spectra count** | **Max score** | **Spectra count** | **Max score** | **Spectra count** | **Max score** |
| **U2 snRNP proteins** | | | | | | | |
|  | **Hsh155** |  |  |  |  |  |  |
| 734 | 325 | 1 | 2.14 |  |  | 1 | 0.92 |
| 745 | 612 |  |  | 2 | 4.21 |  |  |
| 747 | 237 | 1 | 3.39 | 1 | 3.86 |  |  |
| 747 | 612 |  |  | 3 | 5.78 |  |  |
| 778 | 237 | 3 | 5.71 | 1 | 4.03 |  |  |
|  | **Cus1** |  |  |  |  |  |  |
| 747 | 102 |  |  |  |  | 1 | 1.56 |
| 755 | 102 | 4 | 3.67 |  |  |  |  |
| 768 | 48 |  |  |  |  | 1 | 1.42 |
| **NTC proteins** | | | | | | | |
|  | **Clf1** |  |  |  |  |  |  |
| 764 | 668 | 4 | 8.03 |  |  |  |  |
| **RES complex proteins** | | | | | | | |
|  | **Bud13** |  |  |  |  |  |  |
| 323 | 151 |  |  | 1 | 2.03 |  |  |
|  | **Pml1** |  |  |  |  |  |  |
| 669 | 6 |  |  | 1 | 3.33 |  |  |
| **Known splicing proteins** | | | | | | | |
|  | **Cwc21** |  |  |  |  |  |  |
| 52 | 98 | 3 | 8.67 |  |  |  |  |
| 755 | 48 | 1 | 3.74 |  |  |  |  |
|  | **Cwc22** |  |  |  |  |  |  |
| 602 | 530 | 1 | 4.71 |  |  |  |  |
|  | **Prp17** |  |  |  |  |  |  |
| 758 | 375 | 4 | 1.38 |  |  |  |  |

**Figure 6-source data 1a**

| **Prp43_**  **Ntr1GP Residue 1** | **Prp43_**  **Ntr1GP Residue 2** | **Experiment 1** | | **Experiment 2** | | **Experiment 3** | |
| --- | --- | --- | --- | --- | --- | --- | --- |
|  |  | **Spectra count** | **Max score** | **Spectra count** | **Max score** | **Spectra count** | **Max score** |
| 52 | 66 |  |  | 4 | 9.29 |  |  |
| 52 | 71 | 1 | 0.80 |  |  |  |  |
| 66 | 89 |  |  | 1 | 1.01 |  |  |
| 71 | 89 | 10 | 9.51 | 5 | 8.27 |  |  |
| 168 | 182 | 1 | 6.18 | 2 | 2.75 |  |  |
| 237 | 800 | 4 | 5.70 |  |  |  |  |
| 644 | 651 | 5 | 11.71 | 2 | 11.58 | 3 | 7.09 |
| 644 | 745 |  |  | 1 | 1.60 |  |  |
| 644 | 747 | 1 | 2.17 |  |  | 2 | 1.49 |
| 644 | 755 | 10 | 4.63 | 5 | 4.03 |  |  |
| 651 | 755 |  |  | 8 | 7.57 | 1 | 0.28 |
| 663 | 747 |  |  | 4 | 10.92 |  |  |
| 734 | 747 | 9 | 7.07 |  |  | 2 | 1.39 |
| 734 | 764 | 1 | 1.85 |  |  |  |  |
| 734 | 768 | 1 | 4.21 |  |  |  |  |
| 734 | 778 | 24 | 10.70 | 3 | 6.88 | 7 | 6.86 |
| 738 | 745 |  |  | 1 | 3.29 |  |  |
| 738 | 747 |  |  | 9 | 6.90 |  |  |
| 738 | 755 |  |  | 3 | 7.17 |  |  |
| 738 | 764 |  |  | 4 | 5.06 |  |  |
| 738 | 768 | 4 | 6.16 | 6 | 7.21 | 1 | 1.57 |
| 738 | 778 | 19 | 12.14 | 10 | 10.59 | 6 | 6.04 |
| 745 | 755 | 3 | 3.34 | 4 | 5.35 | 2 | 1.67 |
| 747 | 755 | 15 | 10.94 | 18 | 10.55 | 6 | 4.21 |
| 747 | 778 | 12 | 10.02 | 5 | 4.43 | 6 | 2.03 |
| 755 | 764 |  |  | 1 | 3.39 |  |  |
| 755 | 778 |  |  | 4 | 4.24 |  |  |
| 764 | 768 | 2 | 3.91 | 3 | 4.05 |  |  |
| 764 | 775 |  |  | 1 | 6.32 |  |  |
| 764 | 778 |  |  | 2 | 3.90 |  |  |
| 768 | 778 | 10 | 11.37 | 6 | 9.53 | 4 | 4.59 |
| 768 | 785 |  |  | 4 | 6.90 |  |  |
| 768 | 796 |  |  | 2 | 13.91 |  |  |
| 775 | 785 |  |  | 1 | 2.27 |  |  |

**Figure 6-source data 1b**
